# Endovascular cooling is superior to surface cooling in terms of effectiveness by improving the neurological prognosis, but what about the safety?

**DOI:** 10.1186/s13054-020-03005-2

**Published:** 2020-06-01

**Authors:** Patrick M. Honore, Aude Mugisha, Luc Kugener, Rachid Attou, Andrea Gallerani, David De Bels

**Affiliations:** ICU Department, Centre Hospitalier Universitaire Brugmann-Brugmann University Hospital, Place Van Gehuchtenplein 4, 1020 Brussels, Belgium

We read with interest the recent article by Liao et al. who concluded that patients in the endovascular cooling (EC) group had shorter intensive care unit (ICU) hospitalization and a better neurological prognosis than those in the surface cooling (SC) group [1]. In their discussion, they noted several adverse events associated with both techniques including arrhythmia, infection, pneumonia, and bleeding [1]. We would like to speak about a very important and frequent complication linked to EC that the authors neglected to mention. Andremont et al. matched an endovascular cooling catheter cohort of 108 patients with a retrospective historical cohort of 512 patients with femoral venous catheters to compare thrombotic and infectious complications [2]. The duration of catheterization was 4.9 days in the control group vs. 4.2 days in the endovascular cooling group. After propensity score matching, there were significantly more thrombotic complications in the cooling group (12 of 75, 16%) than in the control group (0 of 75, 0%), and 4 patients presented major complications. In another study, Maze et al. investigated the risk of catheter-related thrombosis associated with the use of endovascular cooling catheters in a cohort of 80 patients initially treated with therapeutic hypothermia (TH) of which 61 completed the cooling protocol using an EC device [3]. They further evaluated the incidence of thrombosis between patients on dose-adjusted intravenous unfractionated heparin compared to those on a subcutaneous prophylactic regimen alone. Catheter-related thrombosis was observed in 9/61 (14.7%), with nine events in the prophylaxis group compared to none in the full-dose unfractionated heparin group (22.0% vs. 0.0%). Jung et al. [4] reported a case of an endovascular cooling catheter-related right atrial thrombus (RAT) in a 17-year-old boy treated with therapeutic hypothermia using an endovascular cooling catheter following ventricular fibrillation cardiac arrest. The RAT was detected 3 days after the placement of the cooling catheter and resolved after treatment with enoxaparin for 2 weeks. Thrombosis is an important and potentially life-threatening complication of cooling catheter use, and its prevention with therapeutic anticoagulation may incur significant side effects like bleeding.

## Data Availability

Not applicable.

## Abbreviations

EC: Endovascular catheter
ICU: Intensive care unit
SC: Surface cooling
TH: Therapeutic hypothermia
RAT: Right atrial thrombus
